# A Hole in the Heart, a Hole in the Defenses: A Case of Pseudomonas Endocarditis

**DOI:** 10.7759/cureus.62373

**Published:** 2024-06-14

**Authors:** Azka Naeem, Sajog Kansakar, Arjun Basnet, Muzamil Naeem, Neha Sharma, Saunders Paul, Muhammad H Khan

**Affiliations:** 1 Internal Medicine, Maimonides Medical Center, New York, USA; 2 General Surgery, King Edward Medical University, Lahore, PAK; 3 General Surgery, Lahore General Hospital, Lahore, PAK; 4 Internal Medicine, Gujranwala Medical College, Gujranwala, PAK; 5 Cardiothoracic Surgery, Maimonides Medical Center, New York, USA

**Keywords:** valve replacements, echo cardiogram, end-stage renal disease, mitral valve, pseudomonas aeuginosa, infectious-septic endocarditis

## Abstract

Infective endocarditis (IE) is a rare but serious infection of the cardiac endothelium. This case report presents a rare instance of left-sided *Pseudomonas aeruginosa* endocarditis in an immunocompetent patient without traditional risk factors for IE. *Pseudomonas* endocarditis is uncommon and usually associated with specific factors. The patient in this case was a 30-year-old male with end-stage renal disease, receiving hemodialysis through a tunneled dialysis catheter, who developed a fever. Blood cultures confirmed *P. aeruginosa* as the causative agent, which prompted the administration of appropriate antibiotics and the removal of the catheter. However, subsequent imaging revealed significant damage to the mitral valve. Despite timely mitral valve replacement and aggressive medical treatment, the patient’s condition worsened, and he ultimately succumbed to the infection. This case also emphasizes the necessity of timely diagnosis and intervention. In this patient, by the time it was diagnosed and managed, significant mitral valve damage had already occurred. Therefore, it should be considered a differential diagnosis even in patients with no risk factors and should be managed vigorously. *Pseudomonas* endocarditis is associated with high mortality, and successful treatment often requires a combination of antipseudomonal antibiotics due to the organism’s ability to develop resistance. Surgical intervention, such as valve replacement, is frequently necessary. This case underscores the importance of considering *P. aeruginosa* infection, even in patients without traditional risk factors for IE. Early diagnosis, appropriate antibiotic therapy, and timely surgical intervention are critical for improving outcomes in *Pseudomonas* endocarditis cases.

## Introduction

Infective endocarditis (IE) is a rare but serious infection of the cardiac endothelium that affects approximately 15 out of 100,000 people in the United States annually [1]. The most common organisms responsible for IE are *Staphylococcus aureus*, viridans streptococci, and coagulase-negative staphylococci. However, *Pseudomonas aeruginosa*, an aerobic gram-negative bacillus, can also cause IE, but it is an infrequent cause, accounting for less than 3% of all cases [2]. IE caused by *P. aeruginosa* remains a rare form of IE. Although 95% of cases are associated with injection drug use, healthcare contact is increasingly becoming the primary risk factor. This condition is highly fatal, with a mortality rate of over 73% for patients older than 30 years. It is also strongly associated with prosthetic heart valves and implantable cardiac devices (pacemakers or defibrillators), recent cardiac surgery, left ventricular dysfunction, or immunocompromised states [3]. The most common complications include abscesses in the ring and annulus, congestive heart failure (CHF), embolisms, inability to sterilize valves, splenic abscesses, recurrent bacteremia, and neurologic complications. It can also lead to complications such as myocardial or paravalvular abscess, fistula formation, valve dehiscence, CHF, mycotic emboli, and a mortality rate of between 25% and 40% [4]. Other complications of IE encompass a wide range of issues, including cardiac, metastatic, neurologic, renal, musculoskeletal, and pulmonary complications. Additionally, systemic infection can lead to problems such as embolization, metastatic infection, and mycotic aneurysm. The majority of pseudomonal IEs are right sided; hence, left-sided endocarditis secondary to *P. aeruginosa* in patients with no history of intravenous drug use is a rare entity and typically has a worse prognosis than those with right-sided endocarditis [5]. Hence, we describe a case of left-sided *Pseudomonas *endocarditis in an immunocompetent patient with no risk factors. This case underscores the severity of* P. aeruginosa *endocarditis and the urgency of prompt diagnosis and intervention. Even in patients lacking traditional risk factors, this infection should be considered, demanding proactive management to mitigate significant valve damage.

## Case presentation

**Figure 1:**
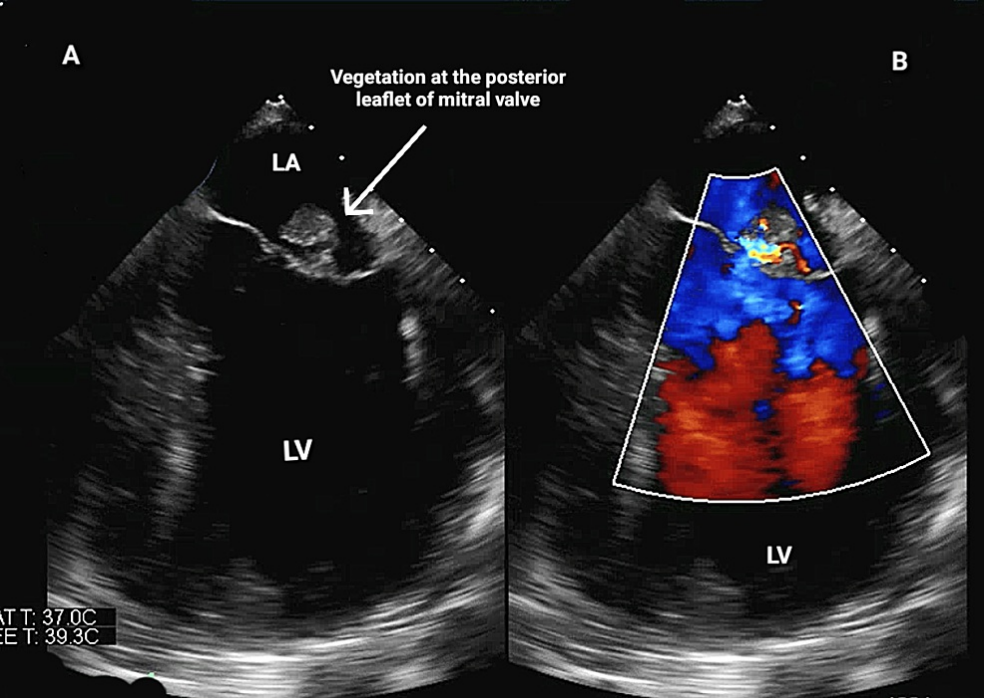
(A) TEE showing vegetation attached to the posterior leaflet of the mitral valve. (B) TEE showing mitral regurgitation with suspicion of mitral valve perforation LA, left atrium, LV, left ventricle; TEE, transesophageal echocardiography

**Figure 2:**
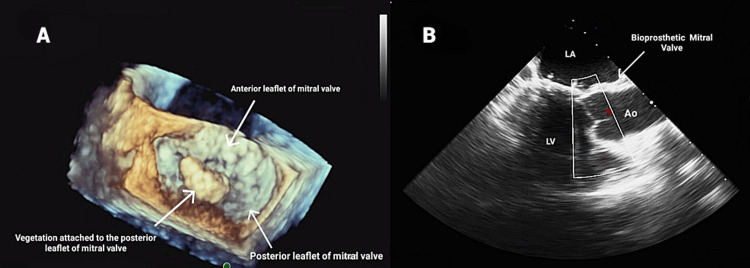
(A) Surgeon’s view of TEE showing vegetation attached to the posterior leaflet of the mitral valve. (B) TEE long axis showing the bioprosthetic mitral valve after replacement Ao, aorta; LA, left atrium; LV, left ventricle; TEE, transesophageal echocardiography

A 30-year-old male, morbidly obese with a past medical history of end-stage renal disease and on hemodialysis (HD) three times a week via a tunneled dialysis catheter (TDC), presented to the hospital with an episode of low-grade fever during his HD session. He was hemodynamically stable at presentation, and his physical examination was within normal limits. The EKG showed sinus tachycardia with T-wave inversions in the lateral leads (I, aVL, V5, V6). Troponin I levels peaked at 3.7 ng/ml, brain natriuretic peptide was 39 pg/ml, and the patient had leukocytosis of 11.9 k/UL. A chest X-ray revealed minimal right basilar opacities. A septic workup was performed, which included blood cultures, C-reactive protein, serological testing for atypical organisms, and erythrocyte sedimentation rate, and the patient was started on empirical treatment with vancomycin, cefepime, and metronidazole. The initial choice of antibiotics was based on providing broad coverage to typical and atypical gram-negative and gram-positive organisms. A blood culture from admission was positive for *P. aeruginosa*. The antibiotic regimen was adjusted to cefepime and amikacin based on sensitivities and infectious disease recommendations. The TDC was removed, and a left internal jugular dialysis catheter was placed to conduct the scheduled HD. A CT of the abdomen and pelvis was done to look for the source of bacteremia, which turned out to be negative. Transthoracic echocardiography (TTE) and transesophageal echocardiography (TEE) revealed a 2.7 × 1.4 cm mitral valve vegetation attached to the atrial side of the posterior mitral valve leaflet with perforation of the mitral valve and eccentric mitral valve regurgitation (Figure 1). The patient’s symptoms improved after antibiotics, and subsequent blood cultures were negative. Cardiothoracic surgery was involved on the same visit given the findings of mitral valve perforation, and the mitral valve was replaced with a bioprosthetic valve (Figure 2). However, the patient did not show any significant improvement in symptoms post-op. Despite aggressive management with appropriate antibiotics and timely replacement of the mitral valve, his clinical condition deteriorated, and his postoperative hospital stay was complicated by persistent acidemia and frequent episodes of inadvertent clot formation during dialysis that led to his demise.

## Discussion

*Pseudomonas* endocarditis is associated with high mortality and specific risk factors, rarely occurring in immunocompetent patients without these risk factors [6]. Symptoms are often nonspecific and may include fever, chills, malaise, night sweats, weight loss, skin rash, or, in some cases, signs of heart failure such as shortness of breath, cough, and leg swelling. Diagnosis is typically confirmed through blood culture, inflammatory markers, echocardiography, and serological testing for atypical organisms such as Q fever, among others [7]. Duke’s criteria, also known as the Modified Duke criteria, are a diagnostic tool used by clinicians to identify infectious endocarditis, an infection affecting the inner lining of the heart or heart valves. The criteria consist of major and minor indicators. Major criteria include positive blood cultures for typical IE-causing organisms and evidence of endocardial involvement seen on echocardiography. Minor criteria include fever, predisposing heart conditions, vascular phenomena like emboli, immunologic phenomena, and microbiological evidence. A definitive diagnosis of IE is made if two major criteria, one major and three minor criteria, or five minor criteria are met. A possible diagnosis is made if there is a combination of one major and one minor criterion or three minor criteria. These criteria aid in standardizing IE diagnosis, which is crucial for a condition with diverse clinical presentations [8]. Blood cultures are typically the first step in diagnosing *Pseudomonas* endocarditis, as they can help identify the type of infection and guide treatment. TTE and TEE are the imaging modalities used to detect valvular vegetation and subsequent valvular complications like annular abscess, valvular perforation, and fistula formation [9].

For IE caused by *P. aeruginosa*, the recommended antibiotic regimen involves high-dose tobramycin (8 mg/kg/day IV or divided doses, given intramuscularly) combined with antipseudomonal penicillin (such as piperacillin, ticarcillin, or azlocillin), or high-dose ceftazidime, cefepime, or imipenem. This treatment should be administered for a duration of six to eight weeks [6]. However, antibiotic therapy alone has shown limited efficacy, particularly in left-sided pseudomonal IE cases. Research suggests that antibiotic concentrations, especially with ceftazidime and tobramycin, often fall below the minimum bactericidal concentration in aortic valve vegetation compared to those in tricuspid valve vegetation. Moreover, the dense microbial population in vegetation may contribute to treatment failure without surgical intervention [6]. Therefore, timely surgical intervention is essential for improved survival rates in left-sided IE caused by *P. aeruginosa*. The treatment of IE secondary to *P. aeruginosa *can be challenging because of the organism’s ability to acquire antimicrobial resistance over time [10]. Beta-lactams have a slow bactericidal effect on *Pseudomonas*, with resistance to the drugs developing rapidly. Hence, the treatment of *P. aeruginosa *endocarditis usually necessitates the combination of antipseudomonal drugs like beta-lactams with aminoglycosides or carbapenems, such as imipenem and meropenem [11]. Successful treatment with antibiotics alone is rare, and adjunctive valvular surgery is indicated for the best chance of survival, especially in cases of refractory endocarditis, persistent bacteremia, or extensive destruction of the valve. Despite aggressive medical and surgical therapy, survival rates can be as low as 38% [12]. Early diagnosis and intervention with antibiotics and surgery are crucial in improving the prognosis of patients with *P. aeruginosa* endocarditis.

In our case, the patient lacked typical risk factors for *Pseudomonas *endocarditis, suggesting a predisposition due to comorbidities and instrumentation. Although antibiotics were initially effective, significant mitral valve damage necessitated surgery. This underscores the limited efficacy of antibiotics alone in combating *Pseudomonas* in IE, emphasizing the crucial role of surgical intervention to manage structural damage and improve outcomes. Despite prompt and evidence-based treatment, the patient succumbed to complications, highlighting the challenges of treating this condition.

## Conclusions

Even in patients without conventional risk factors, suspicion of *P. aeruginosa *infection should remain high. Early diagnosis and treatment are paramount for a favorable prognosis. While dual anti-pseudomonal antibiotics can effectively eradicate bacteremia, surgical intervention is frequently necessary for left-sided IE due to the significant valvular damage caused by *P. aeruginosa*. Antibiotics play a pivotal role in combating bloodstream and endocardium infections during IE. However, their efficacy is often limited, particularly in cases of left-sided IE induced by pathogens like *P. aeruginosa*. This bacterium can rapidly cause damage to heart valves, signifying the urgent need for surgical intervention to manage the condition effectively.

## References

[1] Hassan KS, Al-Riyami D (2012). Infective endocarditis of the aortic valve caused by Pseudomonas aeruginosa and treated medically in a patient on haemodialysis. Sultan Qaboos Univ Med J.

[2] Dawson NL, Brumble LM, Pritt BS, Yao JD, Echols JD, Alvarez S (2011). Left-sided Pseudomonas aeruginosa endocarditis in patients without injection drug use. Medicine (Baltimore).

[3] Ramireddy S, Gudipati S, Zervos M (2020). Expect the unexpected: a rare case of Pseudomonas aeruginosa endocarditis. IDCases.

[4] Chittal AR, Rao SJ, Lakra P, Vietri R, Chawla H (2022). Infective endocarditis from Pseudomonas aeruginosa and group C streptococcus. Cureus.

[5] Hagiya H, Tanaka T, Takimoto K, Yoshida H, Yamamoto N, Akeda Y, Tomono K (2016). Non-nosocomial healthcare-associated left-sided Pseudomonas aeruginosa endocarditis: a case report and literature review. BMC Infect Dis.

[6] Yglesias Dimadi II, Rodríguez Murillo M, Villalobos Zúñiga MA (2023). Infectious endocarditis by Pseudomonas aeruginosa in an immunocompetent adult. Cureus.

[7] Gürtler N, Osthoff M, Rueter F, Wüthrich D, Zimmerli L, Egli A, Bassetti S (2019). Prosthetic valve endocarditis caused by Pseudomonas aeruginosa with variable antibacterial resistance profiles: a diagnostic challenge. BMC Infect Dis.

[8] Topan A, Carstina D, Slavcovici A, Rancea R, Capalneanu R, Lupse M (2015). Assesment of the Duke criteria for the diagnosis of infective endocarditis after twenty-years. An analysis of 241 cases. Clujul Med.

[9] Albloshi AM, Alqumber MA (2021). Infective endocarditis: role of molecular techniques in early diagnosis. Saudi J Biol Sci.

[10] Walczak A, McCarthy K, Paterson DL (2023). A contemporary case series of Pseudomonas aeruginosa infective endocarditis. Medicine (Baltimore).

[11] Aldhaheri K, Andany N, Eshaghi A, Simor AE, Palmay L, Patel SN, Lam PW (2022). Infective endocarditis of a native aortic valve due to Pseudomonas aeruginosa complicated by progressive multi-drug resistance. J Assoc Med Microbiol Infect Dis Can.

[12] Reyes MP, Lerner AM (1983). Current problems in the treatment of infective endocarditis due to Pseudomonas aeruginosa. Rev Infect Dis.

